# An evaluation of the performance of HapMap SNP data in a Shanghai Chinese population: Analyses of allele frequency, linkage disequilibrium pattern and tagging SNPs transferability on chromosome 1q21-q25

**DOI:** 10.1186/1471-2156-9-19

**Published:** 2008-02-27

**Authors:** Cheng Hu, Weiping Jia, Weihua Zhang, Congrong Wang, Rong Zhang, Jie Wang, Xiaojing Ma, The International Type 2 Diabetes 1q Consortium, Kunsan Xiang

**Affiliations:** 1 Shanghai Diabetes Institute, Department of Endocrinology and Metabolism, Shanghai Jiao Tong University Affiliated Sixth People's Hospital, 600 Yishan Road, Shanghai, 200233, China; 2 Section of Cancer Genetics, The Institute of Cancer Research, 15 Cotswold Road, Belmont, Sutton, Surrey, SM2 5NG, UK; 3 Department of Cardiology, Ealing Hospital NHS Trust, Uxbridge Road, Southall, Middlesex, UB1 3HW, UK

## Abstract

**Background:**

The HapMap project aimed to catalog millions of common single nucleotide polymorphisms (SNPs) in the human genome in four major populations, in order to facilitate association studies of complex diseases. To examine the transferability of Han Chinese in Beijing HapMap data to the Southern Han Chinese in Shanghai, we performed comparative analyses between genotypes from over 4,500 SNPs in a 21 Mb region on chromosome 1q21-q25 in 80 unrelated Shanghai Chinese and 45 HapMap Chinese data.

**Results:**

Three thousand and forty-two SNPs were analyzed after removal of SNPs that failed quality control and those not in the HapMap panel. We compared the allele frequency distributions, linkage disequilibrium patterns, haplotype frequency distributions and tagging SNP sets transferability between the HapMap population and Shanghai Chinese population. Among the four HapMap populations, Beijing Chinese showed the best correlation with Shanghai population on allele frequencies, linkage disequilibrium and haplotype frequencies. Tagging SNP sets selected from four HapMap populations at different thresholds were evaluated in the Shanghai sample. Under the threshold of r^2 ^equal to 0.8 or 0.5, both HapMap Chinese and Japanese data showed better coverage and tagging efficiency than Caucasian and African data.

**Conclusion:**

Our study supported the applicability of HapMap Beijing Chinese SNP data to the study of complex diseases among southern Chinese population.

## Background

The International HapMap Project aimed at determining the common patterns of DNA sequence variants, their frequencies, and correlations between them, through genotyping samples from four large populations, Centre d'Etude du Polymorphisme Humain reference individuals from Utah, USA (CEU), Han Chinese in Beijing, China (CHB), Japanese in Tokyo, Japan (JPT), and Yoruba in Ibadan, Nigeria (YRI), at a density of 1 SNP every 5 kb. The populations genotyped in the HapMap can serve as reference populations for the selection of tagging SNPs (tSNPs) that capture most of the variations in the genome. It provides an important shortcut to carry out candidate-gene and genome-wide association studies in a certain population by minimizing the numbers of SNPs need to be genotyped [1-3].

As stated by the International HapMap Consortium, the general applicability of the HapMap data should be confirmed in other populations [1]. Several studies previously performed showed high concordance with HapMap data in allele frequencies and haplotype distributions, and good performance of tSNPs selected from the HapMap SNP data [4-12]. However there are few reports available in the literature which compared the linkage disequilibrium (LD) patterns of the CHB population in the HapMap data with other Chinese populations. Whether the HapMap CHB data can be broadly used in other Chinese populations remained to be a key question.

In our study, over 4500 SNPs from a 21 Mb region on chromosome 1q21-q25 were genotyped in 80 Chinese Hans from Shanghai as a component in the International Type 2 Diabetes 1q Consortium. Located in the southeast of China, Shanghai is over 1,000 kilometers away from Beijing where the CHB samples were recruited. Studies have shown that the Chinese Han population can be geographically divided into two also genetically differential groups, northern Han and southern Han Chinese, separated approximately by the Yangtze River [13-16]. The samples from Shanghai in our study are southern Hans while most of the CHB samples are northern Hans. Although previous studies showed similarity of genetic background between East Asian populations [17,18], no study has directly analyzed the utility of HapMap data in the southern Hans. In our study, we estimated the allele and haplotype frequencies of SNPs in Shanghai individuals and compared them with those provided by the HapMap project. Furthermore, we evaluated the transferability and performance of tSNPs selected from the HapMap data in this Shanghai population.

## Results

### Allele frequencies

**Figure 1:**
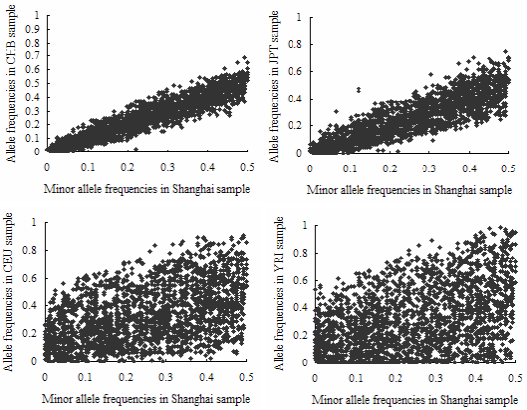
Comparison of allele frequency estimated in Shanghai and four HapMap samples.

We estimated allele frequencies of all SNPs in our Chinese Hans from Shanghai and those in the HapMap populations. The distribution of minor allele frequencies (MAFs) of the SNPs in these five populations is shown in Table 1. By comparing the frequencies of minor alleles defined by our Shanghai sample, we found that they were highly correlated with those from the CHB sample (R = 0.94, *P *< 0.001) (Figure 1). Only 20 (0.66%) SNPs showed an allele frequency difference over 0.15 and no SNP showed difference over 0.2. The allele frequency distribution of 109 SNPs was significantly different between these two groups as shown by the χ^2 ^or Fisher's exact tests (*P *< 0.05). However, by 10,000 permutation tests, only one SNP remained to be significantly different. It was rs12239719 in the *SDHC *gene, with a frequency of 0.02 in the CHB sample vs 0.22 in the Shanghai sample (*P *= 2.28*10^-5^, empirical *P *= 0.0314).

**Table 1:** Minor allele frequency distributions in Shanghai and four HapMap populations.

Population	0	0~0.05	0.05~0.15	0.15~0.25	0.25~0.35	0.35~0.50
Shanghai	0.99%	11.34%	19.10%	19.40%	20.71%	28.47%
CHB	2.85%	9.44%	20.04%	18.95%	19.48%	29.25%
JPT	4.24%	8.12%	20.26%	21.42%	16.61%	29.34%
CEU	1.38%	3.58%	17.71%	21.78%	21.74%	33.80%
YRI	6.06%	8.87%	17.96%	18.56%	19.64%	28.93%

The frequencies estimated from the JPT sample were also highly correlated with the Shanghai sample (R = 0.88, *P *< 0.001). The allele frequency distribution of 547 SNPs was significantly different. By 10,000 permutations, 7 of them remained significant (Additional file 1).

However, the allele frequencies estimated from the CEU and YRI samples differed dramatically from those in the Shanghai sample and Pearson's correlation coefficients between them were 0.46 and 0.41, respectively (*P *< 0.0001) (Figure 1).

### LD structure

The LD structures for the whole region in the Shanghai and the four HapMap samples were shown in Additional file 2. In all non-African populations, the LD structure was similar across populations. In YRI sample, less extent of LD was observed.

**Figure 2:**
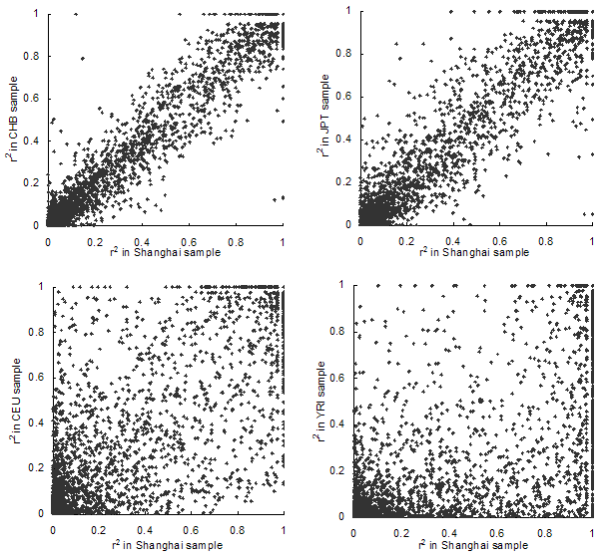
Comparison of r^2 ^of adjacent SNPs in Shanghai and four HapMap populations.

**Figure 3:**
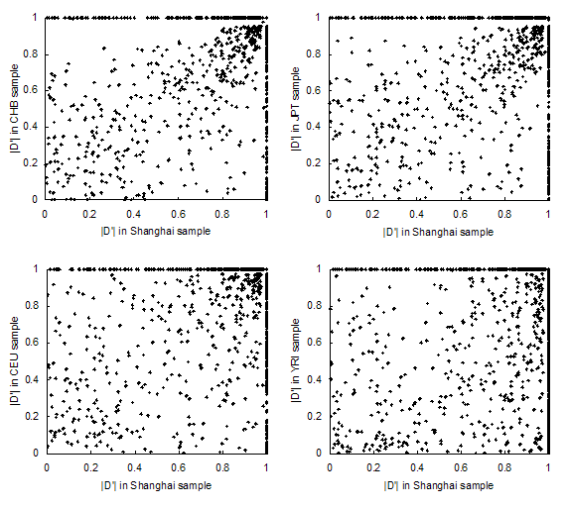
Comparison of |D'| in Shanghai and four HapMap populations.

We measured the extent of pairwise LD between adjacent SNPs by calculating r^2 ^and |D'| and compared them with the corresponding values in the HapMap populations. The correlations between different populations for r^2 ^and |D'| are shown in Figure 2 and 3, respectively. The LD coefficient r^2 ^that estimated from Shanghai samples was highly correlated with that estimated from CHB and JPT samples (CHB: R = 0.9734, *P *< 0.0001; JPT: R = 0.9590, *P *< 0.0001) and less correlated with those estimated from the CEU and YRI samples (CEU: R = 0.8033, *P *< 0.0001; YRI: R = 0.6436, *P *< 0.0001). |D'| calculated from the CHB sample was also most correlated with the Shanghai sample. However, as |D'| appeared to be much more variable, the correlation coefficient R was only 0.5964 (*P *< 0.0001). |D'| calculated from the JPT, CEU and YRI samples were less correlated to that calculated from Shanghai sample (JPT: R = 0.5383, *P *< 0.0001; CEU: R = 0.4945, *P *< 0.0001; YRI: R = 0.3732, *P *< 0.0001).

### Haplotype

A total of 2667, 2648, 2643, 2775 and 2301 SNPs with MAFs greater than 0.05 were included in the analyses of haplotype blocks for the Shanghai, CHB, JPT, CEU and YRI samples, respectively. In the Shanghai sample, 399 blocks were inferred across the region, while 370, 367, 448 and 434 blocks were inferred in the CHB, JPT, CEU and YRI samples. In our samples, the average block size and marker number was 30.51 kb and 5.5 SNPs per block. Similar results were observed in the CHB sample with the average block size of 30.47 kb and the average marker number of 5.48. The JPT and CEU samples also showed similarities to our Shanghai samples to a certain extent. Their average block sizes were 30.77 kb and 29.72 kb and average marker numbers were 5.52 and 5.34 respectively. However, the YRI sample was most distinct from the other populations. Its average block size and marker number were only 18.55 kb and 3.43.

**Figure 4:**
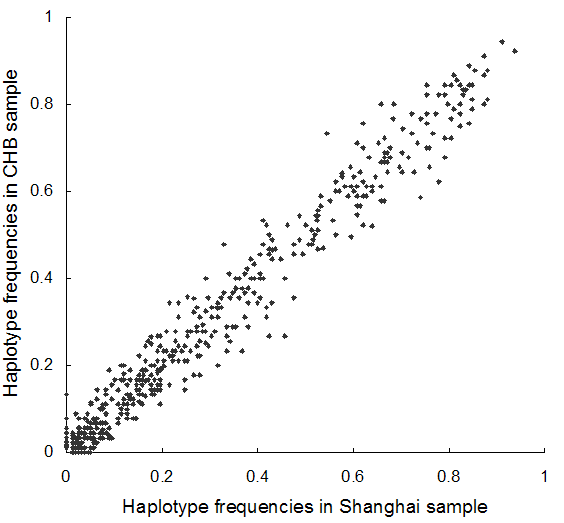
Comparison of haplotype frequencies in Shanghai and CHB.

Between Shanghai and CHB samples, 151 (37.8%) blocks were constructed with the same markers and 589 different haplotypes were observed. Haplotype frequencies were strongly correlated (R = 0.9855, *P *< 0.0001) in these two samples as shown in Figure 4. Only 26 (4.41%) haplotypes showed absolute frequency difference by more than 0.10 and 5 (0.85%) haplotypes by more than 0.15.

### tSNP

To mimic the way an investigator would use the HapMap resource, we used the HapMap populations as reference for tSNP selection at different thresholds and then evaluated the performance of tSNP sets in the Shanghai sample. The performance was determined by computing both the percentage of SNPs captured and the average tagging efficiency. SNPs with MAFs over 0.05 in both the reference population and the Shanghai population were analyzed.

The number and performance of tSNPs selected from four HapMap populations under different thresholds are shown in Table 2. Under the threshold of r^2 ^equal to 0.8 or 0.5, both CHB and JPT samples showed better tagging efficiency than those of CEU and YRI samples in Shanghai Chinese. In the CHB and JPT samples, less than 50% of SNPs were selected as tSNPs and over 93% of variants in the region could be captured in the Shanghai sample. In the CEU samples, 2~3% more SNPs were selected as tSNPs and the tagging efficiency were lower than the CHB and JPT populations. In the YRI samples, we observed the highest coverage but about 50% more SNPs were selected as tSNPs and its tagging efficiency in the Shanghai sample was the lowest among the four populations.

**Table 2:** Performance of tSNP sets selected from the four HapMap populations.

Population		r^2 ^= 0.8			r^2 ^= 0.5		
			
	Number of SNPs analyzed	Number and percentage of tSNPs selected	Percentage of SNPs captured	Tagging efficiency	Number and percentage of tSNPs selected	Percentage of SNPs captured	Tagging efficiency
CHB	2590	1287(49.7%)	0.938	1.889	852(32.9%)	0.669	2.036
JPT	2566	1277(49.8%)	0.932	1.872	820(32.0%)	0.654	2.045
CEU	2474	1272(51.4%)	0.897	1.744	886(35.8%)	0.710	1.982
YRI	2069	1509(72.9%)	0.977	1.340	1184(57.2%)	0.902	1.576

## Discussion

Association study is a common way of identifying the genetic markers for complex diseases, such as diabetes, obesity, cancer, psychiatric illness and cardiovascular disease [19]. The HapMap data provides the LD and tSNP information for four populations to facilitate the design for association studies.

In this study, we evaluated the performance of the HapMap tSNPs in a Shanghai Chinese Han population by comparing allele frequencies, LD patterns, and haplotype frequencies between them. We further assessed transferability of tSNPs selected from the reference populations.

We found that the allele frequencies of the SNPs in this 21 Mb region on chromosome 1q21-q25 were highly correlated between the HapMap CHB and the Shanghai samples. As marker allele frequencies affect the power and sample size required for genetic association studies [20-22], knowing the allele frequencies for the population is important for the design of genetic studies. Our findings indicate that the HapMap CHB sample provides this information for the Chinese population.

We also found the extent of LD were similar among non-African populations. The average haplotype block length, which was dramatically smaller in the YRI sample, was similar in the other populations. R^2 ^values of adjacent SNPs were highly correlated between the CHB and Shanghai samples. However, |D'| was poorly correlated between these two populations. This may be the result of high marker density. With the average marker density of ~7 kb in this study, |D'| values can easily reach their maximum value of 1.0 in one or both populations, causing a ceiling effect.

Not surprisingly, tSNP set selected from the CHB sample performed best in the Shanghai population in terms of coverage and tagging efficiency. Although the JPT sample showed poorer correlation on the allele frequencies and r^2 ^of adjacent SNPs than the CHB sample, the tSNP set selected from the JPT sample performed equally well in this Shanghai population. Also as shown by analyzing ALFRED (the Allele FREquency Database), tSNPs selected from Japanese had high performance in southern Hans [17]. Thus combining JPT and CHB SNP data will be worthy trying when selecting tSNPs from HapMap reference populations.

Several comparative studies have examined similarities and differences between LD patterns and tSNP transferability of HapMap data in various populations based on genes or chromosome regions. North European population was mostly studied among the four HapMap populations. Researchers have performed comparative studies between CEU SNP data and several other populations, including Spanish, Finnish, Estonia and several population isolates [6,8,9,12]. They all came to the same conclusion that the CEU SNP dataset was a robust tool for association studies in these populations. Two studies focused on Asians were also reported. Lim et al analyzed the LD patterns and haplotype structures for ENCODE region ENm010 on chromosome 7p15.2, by genotyping 792 SNPs in 90 healthy Korean individuals. Their analyses showed remarkable similarities in LD strength, haplotype profile, and efficient tSNP transferability among HapMap CHB, JPT and Korean samples [4]. Mahasirimongkol et al analyzed 861 SNPs in 166 drug-related genes between HapMap East Asian populations and Thais. They also found extensive correlation on allele frequency, *Fst *statistics and r^2 ^between these populations [5].

One limitation of this study is that only 79 individuals were analyzed after quality control. Small sample size may bias the allele frequencies estimated in the population, also the extent of LD between markers, and as a result, the portability of tSNPs could be over estimated. It is interesting to note, however, that we didn't detect many significant differences between our sample and the HapMap CHB sample. And as demonstrated previously, for common SNPs with MAFs over 0.05, the use of 60 independent individuals didn't affect the performance of tSNPs significantly [6]. Another limitation is that our samples were composed of normal controls from a case-control study. They were over 65 years old with normal weight and were free from diabetes, hypertension, dyslipidaemia and the family history of these diseases, whereas the HapMap CHB individuals were recruited randomly from Beijing ignoring the disease status. The particularity of our sample might lead to the difference in some SNPs or haplotypes.

## Conclusion

We conclude that the HapMap CHB SNP set has a good portability to the Shanghai population and thus it is a powerful tool for the genetic studies on complex disease in Chinese Hans. Further studies focusing on populations from other regions and nationalities in China are needed to confirm our findings.

## Methods

### Population samples

Eighty unrelated Chinese individuals were recruited from Shanghai Caoyang community as a component of the case-control study in the International Type 2 Diabetes 1q Consortium. All the individuals were normal controls free from diabetes, hypertension or dyslipidaemia. The sample was compased of 55 males and 25 females, aged 74 years in average. This study was reviewed and approved by the institutional review board of Shanghai Jiao Tong University Affiliated Sixth People's Hospital, Shanghai, China. Written informed consents were obtained from all participants.

### SNP selection and genotyping

A 21 Mb region on chromosome 1q21-q25 (position 148.10 Mb to 169.42 Mb, from the NCBI build 35 UCSC genome) was selected for genotyping. Over 4,500 SNPs were genotyped using the Illumina Golden Gate assay (Illumina Inc., San Diego, CA, USA) and the quality control was performed by the 1q Consortium [23]. After removal of SNPs that failed quality control and those not in the HapMap panel, 3,042 SNPs and 79 individuals were analyzed in this study, with an average density of one SNP per 7.0 kb. Detailed SNP information is shown in Additional file 3 and can be also obtained from dbSNP.

The HapMap SNP data of 60 CEU individuals, 45 CHB individuals, 45 JPT individuals and 60 YRI individuals were obtained from HapMap database (release #20).

### Statistical analyses

Allele frequencies were estimated by gene counting and checked for accordance with Hardy-Weinberg equilibrium in each population [24]. Allele frequencies of SNPs were compared between populations by chi-square or Fisher's exact tests, where appropriate. Linkage disequilibrium parameters (|D'| and r^2^) for adjacent SNPs were calculated and haplotype blocks were defined within each population using the confidence interval algorithm [25] and performed by Haploview version 3.32 [26]. Haplotype frequencies were estimated by Expectation – Maximization algorithm [27]. Permutation test that randomly assigns the phenotypes while keeping the genotypes intact was used to obtain empirical *P *values as an alternative to multiple test correction. Pearson's correlation coefficient (R) was used to estimate correlations in allele and haplotype frequencies and linkage disequilibrium parameters among populations. A paired *t*-test was performed to compare the allele frequencies and linkage disequilibrium parameters among the populations using SAS for WINDOWS (version 6.12, SAS Institute Inc., Cary, NC, USA).

SNPs with MAFs over or equal to 0.05 were selected for the analyses of tSNP transferability across populations. The Tagger program in Haploview was used to identify tSNPs that optimally capture allelic variation among SNPs. The tSNPs were selected based on a pairwise approach [28]. An r^2 ^of 0.5 and 0.8 was selected as thresholds for tSNP selection. Coverage of tSNPs was defined as the percentage of SNPs in the evaluated population that had an r^2 ^above 0.5 or 0.8 by the tSNP selected from the reference population. Tagging efficiencies of tSNP sets were defined as the average number of SNPs captured by each tSNP selected.

## Authors' contributions

CH carried out the majority of the analyses and drafted the paper. WJ initialed and supervised data analysis as well as provided the valuable framework to draft the paper. WZ provided helpful comments on data analyses and revised the paper. CW participated in the data analysis. RZ prepared the DNA samples for International Type 2 Diabetes 1q Consortium and participated in the data analysis. JW provided helpful comments and revised the paper. XM recruited the samples and analyzed the clinical data. International Type 2 Diabetes 1q Consortium genotyped the SNPs and performed quality control analyses of the genotype data. KX conceived the study.

## Supplementary Material

Additional File 1 Comparison of allele frequencies between Shanghai and HapMap JPT populations. The data represent the differences of allele frequencies distribution between Shanghai and HapMap JPT populations. Click here for file

Additional File 2 Linkage disequilibrium patterns of 1q21-q25 in Shanghai and four HapMap populations. The data represent the linkage disequilibrium pattern of 1q21-q25 in Shanghai and four HapMap populations. Click here for file

Additional File 3 Information of SNPs analyzed in the study. The data included the rs number, alleles and counts of each genotypes of the SNPs analyzed in the study. Click here for file
