# Supplementary figures and images for: An evaluation of the performance of HapMap SNP data in a Shanghai Chinese population: Analyses of allele frequency, linkage disequilibrium pattern and tagging SNPs transferability on chromosome 1q21-q25
Source: BMC Genet. 2008 Feb 27;9:19. doi: 10.1186/1471-2156-9-19 (PMC2292209; doi:10.1186/1471-2156-9-19)

Shanghai


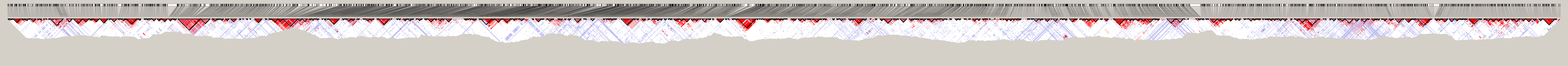


CHB


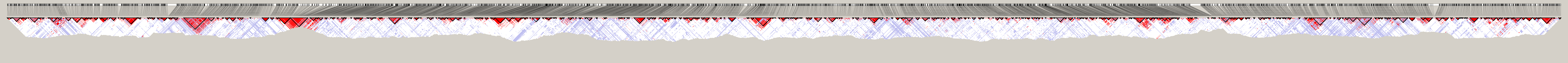


JPT


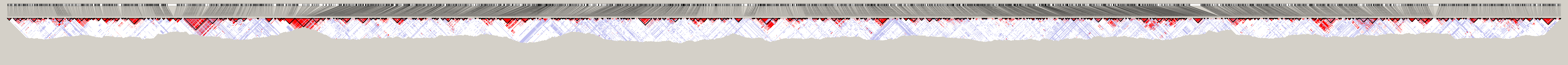


CEU


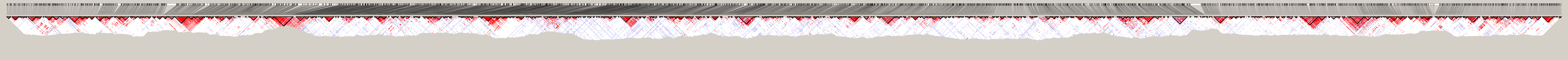


YRI


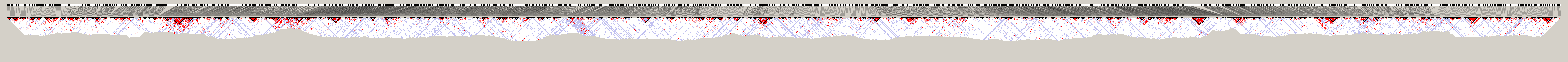

Supplement: Additional File 2 — Linkage disequilibrium patterns of 1q21-q25 in Shanghai and four HapMap populations. The data represent the linkage disequilibrium pattern of 1q21-q25 in Shanghai and four HapMap populations. [file 1471-2156-9-19-S2.doc]
